# Fetal death as an outcome of acute respiratory distress in pregnancy, during the COVID-19 pandemic: a population-based cohort study in Bahia, Brazil

**DOI:** 10.1186/s12884-023-05601-w

**Published:** 2023-05-05

**Authors:** Rita Carvalho-Sauer, Renzo Flores-Ortiz, Maria da Conceição N. Costa, Maria Gloria Teixeira, Ramon Saavedra, Marla Niag, Enny S. Paixao

**Affiliations:** 1 Bahia State Health Department, Núcleo Regional de Saúde Leste, Avenida Esperança, 406. Maria Preta. Santo Antônio de Jesus., 44435-500 Bahia, Brazil; 2 Institute of Collective Health, Federal University of Bahia, Bahia, Brazil; 3 Center for Data and Knowledge Integration for Health (CIDACS), Gonçalo Moniz Institute, Oswaldo Cruz Foundation (FIOCRUZ), Bahia, Brazil; 4 School of Medicine, Federal University of Recôncavo of Bahia, Bahia, Brazil; 5 London School of Hygiene and Tropical Medicine, London, UK

**Keywords:** COVID-19, COVID-19 pandemic, Fetal death, Pregnancy, Pregnancy complications, SARS-CoV-2, Respiratory distress

## Abstract

**Background:**

Fetal loss is one of the most serious adverse outcomes of pregnancy. Since the onset of the COVID-19 pandemic, Brazil has recorded an unprecedented number of hospitalizations of pregnant women due to acute respiratory distress (ARD), thereby, we aimed to assess the risk of fetal deaths associated to ARD during pregnancy in Bahia state, Brazil, in the context of the COVID-19 pandemic.

**Methods:**

This is an observational population-based retrospective cohort study, developed with women at or after 20 weeks of pregnancy, residents in Bahia, Brazil. Women who had acute respiratory distress (ARD) in pregnancy during the COVID-19 pandemic (Jan 2020 to Jun 2021) were considered 'exposed'. Women who did not have ARD in pregnancy, and whose pregnancy occurred before the onset of the COVID-19 pandemic (Jan 2019 to Dec 2019) were considered 'non-exposed'. The main outcome was fetal death. We linked administrative data (under mandatory registration) on live births, fetal deaths, and acute respiratory syndrome, using a probabilistic linkage method, and analyzed them with multivariable logistic regression models.

**Results:**

200,979 pregnant women participated in this study, 765 exposed and 200,214 unexposed. We found four times higher chance of fetal death in women with ARD during pregnancy, of all etiologies (adjusted odds ratio [aOR] 4.06 confidence interval [CI] 95% 2.66; 6.21), and due to SARS-CoV-2 (aOR 4.45 CI 95% 2.41; 8.20). The risk of fetal death increased more when ARD in pregnancy was accompanied by vaginal delivery (aOR 7.06 CI 95% 4.21; 11.83), or admission to Intensive Care Unit (aOR 8.79 CI 95% 4.96; 15.58), or use of invasive mechanical ventilation (aOR 21.22 CI 95% 9.93; 45.36).

**Conclusion:**

Our findings can contribute to expanding the understanding of health professionals and managers about the harmful effects of SARS-CoV-2 on maternal–fetal health and alerts the need to prioritize pregnant women in preventive actions against SARS-CoV-2 and other respiratory viruses. It also suggests that pregnant women, infected with SARS-CoV-2, need to be monitored to prevent complications of ARD, including a careful assessment of the risks and benefits of early delivery to prevent fetal death.

**Supplementary Information:**

The online version contains supplementary material available at 10.1186/s12884-023-05601-w.

## Background

The emergence in December 2019, in China, of the new respiratory-borne virus SARS-CoV-2, the etiologic agent of COVID-19, and its rapid spread across countries and continents resulted in the worst pandemic of the twenty-first century [1, 2]. Over the first three years of this health crisis, the world has experienced the dramatic loss of more than 6,696,926 lives due to this disease [3]. Far beyond a health tragedy, the disruptive effect of the COVID-19 pandemic on the social and economic structures worldwide has disproportionately affected the most vulnerable populations [1, 4, 5].

In this pandemic context, many pregnant women experienced greater social vulnerability, besides the higher susceptibility to complications of viral infections [6]. The health and well-being of pregnant women and their fetuses have been affected, and the advances in women's health, hard-won over decades, have been threatened [7]. Not by chance, in August 2020, the Pan American Health Organization issued an alert for member countries to intensify efforts to ensure access to prenatal care and actions to reduce maternal and perinatal morbidity and mortality [8].

In Brazil, a middle-income country, until December 31, 2022, more than 36,331,281 cases and 693,853 deaths due to SARS-CoV-2 infection were reported, corresponding to a cumulative incidence and mortality of 17,288.5 and 330,2 (per 100,000 inhabitants), respectively, and a case fatality rate of 1.9% [9]. This country is among the first in number of cases and deaths from COVID-19 in the world [3].

Furthermore, since the onset of the COVID-19 pandemic, Brazil has recorded an unprecedented number of hospitalizations of pregnant women due to acute respiratory distress (ARD). Epidemiological surveillance data showed that in 2020 and 2021, there were 28,238 reports of ARD in pregnant women in Brazil, compared to 2,252 reported in 2018–2019 [10].

Despite this worrying scenario, there are still few studies evaluating the effects of the COVID-19 pandemic on gestational outcomes, particularly in developing countries, where the spread of SARS-CoV-2 adds to the worsening of social inequalities and problems in the health system [11].

Fetal loss is one of the most serious adverse outcomes of pregnancy [12]. So, we aimed to assess the chance of fetal death associated with acute respiratory distress during pregnancy, in the context of the COVID-19 pandemic in Bahia, Brazil.

## Methods

### Study design, location, and period

We performed a population-based retrospective cohort study, using administrative data on live births, fetal deaths, and ARD in pregnant women, from January 2019 to June 2021, in Bahia, a state with 14,985,284 inhabitants located in the Northeast region of Brazil [13].

### Study population

The population comprised women with ≥ 20 weeks of pregnancy, residents in Bahia state. Therefore, the outcome "fetal deaths" in this study included spontaneous intrauterine death of fetuses at or after 20 gestational weeks (or weighing ≥ 500 g, or length ≥ 25 cm, if unknown gestational age [14]).

During the COVID-19 pandemic, there was a significant increase in maternal mortality from several causes in Brazil [15, 16]. As this study focuses on the association between ARD during pregnancy and fetal death, we chose not to include fetal deaths that occurred concurrently with maternal deaths, as it would be more difficult to determine clearly whether fetal death occurred before or after maternal death, in these cases.

The exposed group consisted of women who had a pregnancy ending in 2020 or 2021, and who had a history of hospitalization due to acute respiratory distress during this pregnancy, consistent with respiratory viruses, that is, a combination of flu-like symptoms such as fever and cough with acute respiratory distress syndrome, and/or dyspnea, and/or O_2_ saturation < 95% in ambient air, and/or cyanosis, and/or hypotension and/or acute breathing insufficiency, regardless of its etiology [17].

The unexposed group was women who had a pregnancy ending in 2019 (pre-pandemic period) and did not undergo a hospital stay due to respiratory distress, because they were pregnancies free of the possible effects of SARS-CoV-2 infection and the environmental peculiarities of the COVID-19 pandemic period.

### Data sources

We used data from robust health information systems [18], such as the Live Births Information System / *Sistema de Informação sobre Nascidos Vivos* (SINASC); the Mortality Information System / *Sistema de Informação sobre Mortalidade* (SIM); and the Acute Respiratory Syndrome Surveillance System / *Sistema de Vigilância Epidemiológica da Síndrome Respiratória Aguda Grave* (SIVEP). In Brazil, births, deaths, and respiratory distress consistent with respiratory viruses are under universal surveillance and mandatory registration in all public and private health services.

SINASC and SIM contain all national records of births and deaths, respectively, and their coverages are approximately 100% [19–21]. Death records from Brazil are classified in the same quality category as some developed countries such as Germany, France, Italy, Norway, Netherlands and Spain [22].

SIVEP contains all national records of people who present ARD consistent with respiratory viruses, reported by health services. But, if it occurs outside a health service, such as death from ARD at home, epidemiological surveillance teams carry out an epidemiological investigation and reporting at SIVEP [17, 23].

Thus, except for the possibility of underreporting, SINASC, SIM, and SIVEP contain, respectively, all records of births, deaths, and acute respiratory syndromes that occurred in the Brazilian population.

### Data linkage process

Maternal data from 479,038 reports of live births, 333 maternal deaths and 6,841 fetal deaths, and data from 13,077 reports of ARD in women aged 9 to 49 years, from January 1, 2019, to June 30, 2021, were obtained from SINASC, SIM, and SIVEP, respectively. We performed a probabilistic linkage between data of those three systems, using as binding keys the full name, date of birth, and the municipality of residence. Records with at least 85% of similarity were filtered. So, we performed a manual review, with a comparison of home addresses, to confirm only valid pairs.

Probabilistic linkage was done with the fastLink package [24], in the R software [25].

### Variables

The study variables, related to the sociodemographic characteristics of the participants, were: Age group, in years (19 or less, 20—35, 36 or more); Education, in years (0—3, 4—7, 8—11, 12 or more); Race Color (White, Black, Mixed, Oriental, Indigenous); Marital Status (Single or Divorced or Widow, Married or Stable Union) and Human Development Index of municipality of residence (High or Very High, Medium, Low or Very Low).

The variables related to the characteristics of the pregnancy were: Fetal death (Yes, No); Acute Respiratory Distress during pregnancy (Yes, No); Type of pregnancy (Single fetus, Twin fetus or more); Fetus sex (Male, Female); Parity (1st pregnancy, 2nd or more); Type of delivery (Vaginal, Cesarean); Congenital anomaly (Yes, No); and Gestational age, in weeks (1st quartile, median, 3rd quartile).

### Statistical analyzes

To examine the association between ARD in pregnancy and fetal death, we initially performed a descriptive analysis of the data, in which the study participants were divided into two groups, according to their history of ARD during pregnancy (Yes / No). The sociodemographic and gestational characteristics of the participants (age group, education, race/color, marital status, Human Development Index (HDI) of the municipality of residence, fetal death as outcome of pregnancy, type of pregnancy, fetus sex, parity, type of delivery and congenital anomaly of fetus) were described by the absolute and relative frequencies of each variable. The variable gestational age, in weeks, was verified by quartiles.

Chi-squared test and Two-tailed Z-test, for comparing proportions, was performed to identify divergences in sociodemographic and gestational characteristics between women with and without a history of ARD during pregnancy. We considered a significance level of 5%.

We applied a bivariate logistic regression model to verify the risk of fetal death after ARD during pregnancy and identify potential confounders. Those variables that showed association with statistical significance in bivariate analysis were included in a multiple model, to estimate the adjusted Odds Ratio (OR) with 95% confidence intervals (CI).

To quantify the risk of fetal death after ARD in pregnancy, specifically caused by laboratory-confirmed COVID-19, this same analysis was repeated, keeping in the exposed group only participants with reverse-transcriptase polymerase chain reaction (RT-PCR), antigen detection, or IgM serology tests positive for SARS-CoV-2 infection. We also assessed the risk of fetal death after ARD in pregnancy with Intensive Care Unit (ICU) admission, with invasive mechanical ventilation, and with vaginal or cesarean delivery.

All analyzes were conducted with R v. 4.1.3 [25]. This study is reported in accordance with the STROBE statement [26] (Supplementary STROBE Check-list).

## Results

**Fig. 1:**
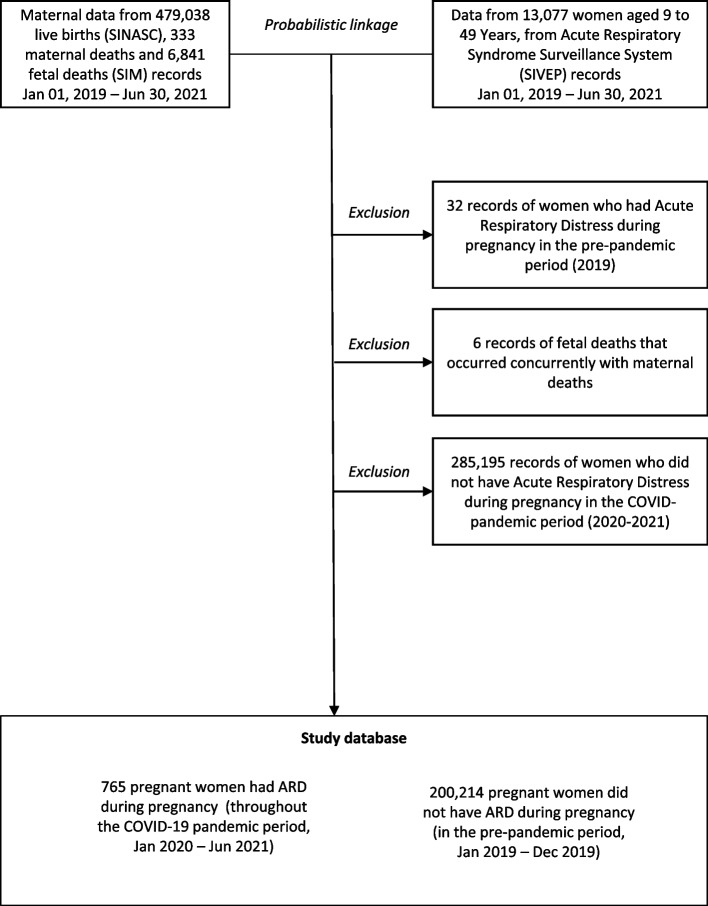
Flowchart of the database composition for the study 'Fetal death as an outcome of acute respiratory distress in pregnancy, during the COVID-19 pandemic: a population-based cohort study in Bahia, Brazil'. Source: Elaborated by the authors. SINASC = Live Births Information System; SIM = Mortality Information System; SIVEP = Acute Respiratory Syndrome Surveillance System

After probabilistic linkage of data from SINASC, SIM and SIVEP, 765 participants were identified as pregnant women who had ARD during pregnancy (throughout the COVID-19 pandemic, Jan 2020 – Jun 2021), and another 200,214 who did not (Jan to Dec 2019). The 32 participants who had ARD during pregnancy in the pre-pandemic period (2019); 6 (six) fetal deaths that occurred concurrently with maternal deaths, and the 284,862 women who did not have ARD during pregnancy in the COVID-19 pandemic period (until Jun, 2021) were excluded (Fig. 1).

Both groups of participants, with and without a history of ARD during pregnancy, were mainly women aged between 20 and 35 years (65.9% and 70.1%), with incomplete secondary education (59.3% and 62.0%), mixed race (75.5% and 76.6%), and single or without a partner (64.5% and 53.7%), respectively. Among women with no history of ARD during pregnancy, there was a higher proportion of residents in municipalities with medium and low HDI (Table 1).

**Table 1:** Acute Respiratory distress (ARD) in pregnant women (N and %) according to sociodemographic characteristics. Bahia, Brazil, Jan 2019—Jun 2021 ^a^

	**ARD during pregnancy**				
**Sociodemographic Characteristics**	**Yes (** ***N*** ** = 765)**		**No (** ***N*** ** = 200,214)**		***p ***^***b***^
	**n**	**%**	**n**	**%**	
*Age group (years)*	*762*	*100.00*	*199,993*	*100.00*	*0.245*
≤ 19	98	12.86	34,140	17.07	
20 – 35	502	65.88	140,204	70.10	
≥ 36	162	21.26	25,649	12.82	
*Education (in years)*	*700*	*100.00*	*191,853*	*100.00*	*0.869*
0 to 3	25	3.57	6,463	3.37	
4 to 7	126	18.00	38,151	19.89	
8 to 11	415	59.28	118,881	61.96	
12 or more	134	19.14	28,358	14.78	
*Race / Color*	*711*	*100.00*	*185,187*	*100.00*	*0.983*
White	55	7.74	14,794	7.99	
Black	112	15.75	26,359	14.23	
Mixed	537	75.53	142,339	76.86	
Oriental	6	0.84	819	0.44	
Indigenous	1	0.14	876	0.47	
*Marital status*	*693*	*100.00*	*192,639*	*100.00*	*0.157*
Single, divorced or widow	447	64.50	103,374	53.66	
Married or stable union	246	35.50	89,265	46.34	
*HDI of the municipality of residence*	*765*	*100.00*	*200,175*	*100.00*	***0.002***
High or Very High (≥ 0.700)	310	40.52	55,601	27.78	
Medium (0.600—0.699)	313	40.92	60,717	30.33	
Low or Very Low (≤ 0.599)	142	18.56	83,857	41.89	

Sources: Live birth information system (SINASC); Mortality information system (SIM); Epidemiological Surveillance System of Acute Respiratory Distress (SIVEP); Brazilian Institute of Geography and Statistics (IBGE)

*HDI* Human Development Index

^a^The exposed group (ARD during pregnancy = Yes) is composed of women who had ARD in pregnancy during the COVID-19 pandemic period (Jan 2020 to Jun 2021). The non-exposed group (ARD during pregnancy = No) comprised women who had a pregnancy before the onset of the COVID-19 pandemic (Jan to Dec 2019) and did not have ARD during pregnancy. These groups differ not only because of ARD during pregnancy but also because of the environmental changes resulting from the COVID-19 pandemic

^b^Chi-square teste (significance level of 5%)

The number in each category varies due to the presence of missing data

The proportions of sociodemographic variables were similar for participants who were and were not exposed to ARD during pregnancy, except for the HDI of their municipality of residence (*p* = 0.002), which showed a statistically significant difference (Table 1).

Women exposed to ARD during pregnancy had the median (and interquartile range) gestational age of symptom onset at 33 weeks (27; 37 weeks); among these, those that resulted in fetal death and live birth, respectively, were 31 (25; 36) and 38 (36; 39) weeks pregnant at the time of delivery. On the other hand, pregnancies not exposed to ARD, but which resulted in stillbirth, had a median (and interquartile range) duration of 36 (26; 37) weeks. And for pregnancies not exposed to ARD, and resulting in a live birth, the duration was 39 (38; 40) weeks (data not shown in table).

The percentage of fetal deaths (*p* < 0.001), twin pregnancies (*p* < 0.001) and cesarean deliveries (*p* < 0.001) was higher among women with a history of ARD during pregnancy, compared to those without. On the other hand, there was no statistically significant difference in the proportions of congenital anomaly (*p* = 0.123), fetus sex (*p* = 0.410), and maternal parity (*p* = 0.169) between the groups of women with and without exposure to ARD during pregnancy (Table 2).

**Table 2:** Acute Respiratory Distress (ARD) in pregnant women (N and %) according to gestational characteristics. Bahia, Brazil, Jan 2019 – Jun 2021 ^a^

	**ARD during pregnancy**				
**Gestational characteristics**	**Yes (** ***N*** ** = 765)**		**No (** ***N*** ** = 200,214)**		***p ***^***b***^
	**n**	**%**	**N**	**%**	
*Fetal death*	*765*	*100.00*	*200,214*	*100.00*	** < ** ***0.001***
Yes	32	4.18	2,678	1.34	
No	733	95.82	197,536	98.66	
*Type of pregnancy*	*695*	*100.00*	*199,803*	*100.00*	** < ** ***0.001***
Single fetus	663	95.40	195,527	97.86	
Twin fetus or more	32	4.60	4,276	2.14	
*Fetus sex*	*697*	100.00	*200,001*	*100.00*	*0.410*
Male	345	49.50	102,263	51.13	
Female	352	50.50	97,738	48.87	
*Parity*	*618*	*100.00*	*179,053*	*100.00*	*0.169*
1^st^ pregnancy	199	32.20	62,522	34.92	
2^nd^ or more	419	67.80	116,531	65.08	
*Type of delivery*	*694*	*100.00*	*199,972*	*100.00*	** < ** ***0.001***
Vaginal	234	33.72	108,256	54.14	
Cesarean	460	66.28	91,536	45.77	
*Congenital anomaly*	*645*	100.00	*193,458*	*100.00*	*0.123*
Yes	9	1.40	1,512	*0.78*	
No	636	98.60	191,946	*99.22*	

Source: Live birth information system (SINASC); Mortality information system (SIM); Epidemiological Surveillance System of Acute Respiratory Distress (SIVEP)

^a^The exposed group (ARD during pregnancy = Yes) is composed of women who had ARD in pregnancy during the COVID-19 pandemic period (Jan 2020 to Jun 2021). The non-exposed group (ARD during pregnancy = No) comprised women who had a pregnancy before the onset of the COVID-19 pandemic (Jan to Dec 2019) and did not have ARD during pregnancy. These groups differ not only because of ARD during pregnancy but also because of the environmental changes resulting from the COVID-19 pandemic

^b^Two-tailed Z test (significance level of 5%)

All 765 pregnant women who had ARD were hospitalized, 286 (37.38%) were admitted to the ICU and 127 (16.6%) received invasive ventilation. SARS-CoV-2 was identified as the etiologic agent of ARD in 432 (56.47%) cases (379 confirmed by laboratory tests, and 53 by imaging exams and clinical-epidemiological criteria), while 10 (1.30%) cases were due to other respiratory viruses. Also, 323 (42.23%) cases of ARD during pregnancy had no etiologic diagnosis (data not shown in tables).

In the multiple logistic regression model, the chance of fetal death was 306% higher in women exposed to ARD during pregnancy (aOR 4.06 CI 95% 2.66; 6.21). In addition, women aged 36 or over (aOR1.57 CI 95% 1.34; 1.84), with schooling from 0 to 3 (aOR 4.17 CI 95% 3.41; 5.10) and from 4 to 7 (aOR 1.72 CI 95% 1.45; 2.05) years of study, with twin pregnancy (aOR 3.88 CI 95% 3.20; 4.70), with male fetus (aOR 1.19 CI 95% 1.09; 1.31) and with cesarean deliveries (aOR 0.43 CI 95% 0.38;0.47) were independently associated with fetal deaths (Table 3).

**Table 3:** Crude and adjusted odds ratio obtained through logistic regression analysis for the association between Acute Respiratory Distress (ARD) during pregnancy and fetal deaths. Bahia, Brazil, Jan 2019—Jun 2021 ^a^

**Covariates**	**Crude Odds Ratio**^b^	**CI 95%**	**Fully adjusted Odds Ratio **^**c**^	**CI 95%**
*ARD during pregnancy*				
No	1	-	1	-
Yes	**3.22**	**2.26;4.60**	**4.06**	**2.66;6.21**
*Age group (years)*				
≤ 19	1	-	1	-
20 – 35	**0.89**	**0.81;0.99**	1.12	0.99;1.27
≥ 36	**1.34**	**1.18;1.54**	**1.57**	**1.34;1.84**
*Education (in years)*				
12 or more	1	-	1	-
08 – 11	**1.22**	**1.05;1.41**	1.11	0.95;1.31
4 – 7	**2.02**	**1.72;2.37**	**1.72**	**1.45;2.05**
0 – 3	**6.14**	**5.13;7.36**	**4.17**	**3.41;5.10**
*Type of pregnancy*				
Single fetus	1	-	1	-
Twin fetus or more	**3.11**	**2.64;3.66**	**3.88**	**3.20;4.70**
*Fetus sex*				
Female	1	-	1	-
Male	**1.18**	**1.09;1.27**	**1.19**	**1.09;1.31**
*Type of delivery*				
Vaginal	1	-	1	-
Cesarean	**0.44**	**0.39;0.47**	**0.43**	**0.38;0.47**
*HDI on the municipality of residence*				
High or Very High (≥ 0.700)	1	-	1	-
Medium (0.600—0.699)	**1.16**	**1.05;1.28**	0.99	0.89;1.11
Low or Very Low (≤ 0.599)	**1.25**	**1.13;1.39**	0.97	0.86;1.10

Source: Live birth information system (SINASC); Mortality information system (SIM); Epidemiological Surveillance System of Acute Respiratory Distress (SIVEP); Brazilian Institute of Geography and Statistics (IBGE)

*HDI* Human Development Index

^a^The exposed group (ARD during pregnancy = Yes) is composed of women who had ARD in pregnancy during the COVID-19 pandemic period (Jan 2020 to Jun 2021). The non-exposed group (ARD during pregnancy = No) comprised women who had a pregnancy before the onset of the COVID-19 pandemic (Jan to Dec 2019) and did not have ARD during pregnancy. These groups differ not only because of ARD during pregnancy but also because of the environmental changes resulting from the COVID-19 pandemic

^b^Bivariate analysis

^c^Adjusted for ARD during pregnancy, age group, education, fetus sex, type of pregnancy, type of delivery, and HDI of the municipality of residence of pregnant women

The sensitivity analysis, restricted to COVID-19 laboratory-confirmed cases, showed that the risk of fetal death was 345% higher (aOR 4.45 CI 95% 2.41; 8.20) when compared to the unexposed group and controlled for maternal age group, maternal education, type of pregnancy, sex of the fetus and type of delivery (Table 4). Also, women who had ARD during pregnancy accompanied by vaginal delivery (aOR 7.06 CI 95% 4.21; 11.83), or by ICU admission (aOR 8.79 CI 95% 4.96; 15.58), or by use of invasive mechanical ventilation (aOR 21.22 CI 95% 9.93; 45.36), had the chance of fetal deaths lifted, controlled for the same covariables (Table 4). ARD during pregnancy combined with cesarean delivery (aOR 1.03 CI 95% 0.91; 1.17) was not associated with fetal deaths (data not show in Table).

**Table 4:** Crude and adjusted odds ratio obtained through logistic regression analysis for the association between Acute Respiratory Distress (ARD) during pregnancy (and COVID-19 laboratory confirmation, or UCI admission, or invasive mechanical ventilation, or vaginal delivery) and fetal death. Bahia, Brazil, Jan 2019 – Jun 2021 ^a^

**Variables**	**Crude Odds Ratio** ^b^	**CI 95%**	**Fully adjusted Odds Ratio** ^c^	**CI 95%**
No history of ARD during pregnancy	1	-	1	-
ARD during pregnancy due to COVID-19, laboratory criteria	**3.04**	**1.81; 5.10**	**4.45**	**2.41; 8.20**
ARD during pregnancy, with Intensive Care Unit admission	**5.54**	**3.51; 8.75**	**8.79**	**4.96; 15.58**
ARD during pregnancy, with invasive mechanical ventilation	**6.99**	**3.76; 12.99**	**21.22**	**9.93; 45.36**
ARD during pregnancy, with vaginal delivery	**6.58**	**4.36; 9.93**	**7.06**	**4.21; 11.83**

^a^The exposed group (ARD during pregnancy = Yes) is composed of women who had ARD in pregnancy during the COVID-19 pandemic period (Jan 2020 to Jun 2021). The non-exposed group (ARD during pregnancy = No) comprised women who had a pregnancy before the onset of the COVID-19 pandemic (Jan to Dec 2019) and did not have ARD during pregnancy. These groups differ not only because of ARD during pregnancy but also because of the environmental changes resulting from the COVID-19 pandemic

^b^Bivariate analysis

^c^Adjusted for age group, education (in years), type of pregnancy (single or twin fetuses), fetus sex, type of delivery (vaginal or cesarean)

## Discussion

This study showed that during the first two years of the COVID-19 pandemic (2020–2021) SARS-CoV-2 virus was the main cause of ARD in pregnant women from Bahia, Brazil. Also, it was possible to identify that most cases of ARD in pregnancy occurred in the third trimester, and that the median length of gestation of women who had ARD with fetal death was much shorter (-8 weeks) than that of women who did not have ARD nor fetal death in pregnancy.

Not surprisingly, ARD during pregnancy was strongly associated with fetal deaths, and the greater the clinical severity, the greater was the chance of fetal loss.

ARD is known to be a multietiological condition with significant morbidity and mortality that, in pregnant women, promotes maternal hypoxia and acute fetal distress due to reduced oxygen supply [27, 28]. Generally, pregnant women are less tolerant of hypoxemia and more prone to complications from viral infections [29, 30], such as secondary bacterial infection, disseminated intravascular coagulation, and multi-organ failure, and this critical maternal illness can lead to serious consequences for the fetus [31].

No wonder our results showed that a large part of the participants diagnosed with ARD required intensive care, and this reflected in a higher risk of fetal loss. The chance of fetal death was more than four times higher among the exposed compared to the unexposed group, either for all etiologies or specifically when the causative agent of ARD was the SARS-CoV-2 virus. And critical forms of ARD, requiring artificial respiration support, increased the chance of this outcome by up to 21 times.

SARS-CoV-2 can threaten the health of pregnant women and their fetuses in different ways. First, this virus disturbs cardiovascular homeostasis, by interfering with the signaling of the Renin-Angiotensin System (RAS), which may be related to hypertensive disorders of pregnancy, reduced placental blood flow by vasoconstriction, and poor fetal outcomes [32, 33]. Data from the INTERCOVID prospective cohort [34] showed that COVID-19 during pregnancy is strongly associated with preeclampsia, regardless of other risk factors and pre-existing conditions. Pre-eclampsia, in turn, is associated with fetal deaths [35].

Moreover, in pregnancy, there is a physiological elevation in thrombin and fibrinogen, while a decrease in anti-clotting and fibrinolytic activities [36]. In COVID-19, a state of hypercoagulability occurs due to RAS dysregulation and immune response mechanisms [37, 38]. Then, COVID-19 in pregnancy can be considered a synergism of pro-coagulation states, lifting the risks of maternal coagulopathy and placental lesions, with worse consequences for the fetus [39, 40].

The placenta is also a target site for SARS-CoV-2 acute damage [40]. SARS-CoV-2 infection causes a type of placentitis characterized by three patologic lesions: chronic histiocytic intervillositis, massive perivillous fibrin deposition and trophoblast necrosis; and this inflammatory condition can promote placental malperfusion and insufficiency and be lethal to the fetus [41–43]. Apart from this, in SARS-CoV-2 infection, the unique characteristics of the pregnant woman's immune system make them vulnerable to cytokine storm, an excessive immune response that impairs the prognosis of this disease to the mother and fetus [38, 40]

Although several respiratory viruses can cause ARD, more than half of the cases in our study were diagnosed with SARS-CoV-2 infection. Only 1.3% were due to other respiratory viruses, and 41.17% did not have an etiological diagnosis. This last information discloses flaws in the health system, like problems accessing diagnostic exams, or the loss of opportunity to accomplish laboratory tests in a timely manner. However, it is very likely that most of these undiagnosed cases were also caused by SARS-CoV-2, given the 1154% increase in the frequency of ARD in pregnant women in 2020 and 2021 [10], compared to the years before the COVID-19 pandemic, when all other respiratory viruses were already circulating. Furthermore, the results of the regression models were very similar when we analyzed all ARD cases and only those with laboratory diagnoses of SARS-CoV-2 infection.

Some studies that evaluated participants with a history of COVID-19 during pregnancy observed no increase in fetal deaths [44–46]; these studies included pregnant women with all clinical presentations of this infection, with the vast majority of participants having asymptomatic, mild or moderate forms. On the other hand, in our study, the exposed participants had a more severe presentation (acute respiratory distress).

Our findings are supported by other studies that involved women with severe COVID-19 during pregnancy. In Sweden, Norway and Denmark, Örtqvist and colleagues (2023) [47] observed that the percentage of stillbirths in women admitted to the ICU due to severe COVID-19 was 14 times higher than that for all birthing women (4.2% versus 0.3%, respectively). In the United Kingdom, Vousden et al. (2022) [48] found a higher risk of stillbirth (aOR 2.51, 95% CI 1.35–4.66) among pregnant women with severe COVID-19, than those with mild to moderate symptoms of this infection. However, our study showed a more pronounced effect. This was probably because our comparison group (not exposed) included only women who were pregnant before the onset of the COVID-19 pandemic, not exposed to SARS-CoV-2 or the disruptions that occurred in health services during the COVID-19 pandemic.

We adjusted our results for important variables associated with fetal deaths. In our study maternal age ≥ 36 years, low level of education (0 – 7 years of schooling), twin pregnancies, fetal sex, and cesarean delivery were independently associated with fetal deaths, which corroborates the literature, as age greater than 35 years is a known risk factor for adverse obstetric outcomes [49] and more severe forms of COVID-19 [50]. Low schooling is related to worse conditions of housing, employment, income, gender violence, neighborhood, and other social determinants of health [51], and the COVID-19 pandemic may have exacerbated these social vulnerabilities. The risk of fetal death in twin pregnancies is usually higher than in singleton pregnancies [52]; but in pregnant women with ARD, the high metabolic demand of multiple fetuses can determine an even poorer prognosis. Regarding fetal sex, we found a higher chance of death for male fetuses, which is a finding reported by several authors over decades and suggests a greater prenatal vulnerability of males [53–55].

It is relevant to highlight that ARD during pregnancy combined with cesarean delivery was not associated with fetal deaths; but cesarean delivery, singly, demonstrated a protective effect against this adverse gestational outcome. In fact, when correctly indicated, cesarean delivery can benefit the ill pregnant woman, and save the fetus in acute distress, depending on the gestational age and fetal maturity. However, it is necessary to consider that urgent cesarean preterm deliveries can prevent fetal death, but studies are needed to assess the survival of premature neonates in these situations.

Although there is no information about the vaccination status of the participants of this study, it is likely that most of them were not immunized against SARS-CoV-2, since the COVID-19 vaccination of pregnant women started in Bahia in May 2021 [56], only for pregnant women with comorbidities. For pregnant women without comorbidities, this vaccination began in July 2021 [57], but this study included only pregnancies that ended by June 30^th^ of that same year.

Our results must be interpreted considering their strengths and limitations. Limitations of this study include the use of administrative data, the use of preliminary data for 2021, the possibility of errors in the linkage of the databases, lack of knowledge regarding vaccine status, virus variants, maternal comorbidities, and potential underreporting. Strengths include using data of events under mandatory and universal surveillance (births, deaths, and acute respiratory distress) from robust and well-consolidated health information systems, and the controlling for important confounders. To the best of our knowledge, this is the first study to estimate the risk of fetal deaths associated to ARD in pregnancy, in the context of COVID-19 pandemic, in a middle-income country, and in a statewide population-based cohort.

## Conclusion

Our results reiterate observations from previous studies that the mother-infant binomial is very vulnerable to the deleterious effects of severe COVID-19. It has important implications for public policies and clinical practice, as it alerts the need to prioritize pregnant women in preventive actions against SARS-CoV-2 and other respiratory viruses. Our study also suggests that pregnant women infected with SARS-CoV-2 need to be monitored to avoid complications of ARD, including a careful assessment of the risks and benefits of early delivery to prevent fetal death.

New studies are needed to determine the mechanisms by which severe COVID-19 in pregnant women can lead to fetal death and the influence of environmental issues such as system constraints, co-infection with other respiratory viruses, delays in access to diagnosis and assistance, with the increased risk of fetal losses.

## Supplementary Information

**Additional file 1.** 

**Additional file 2.** 

## Data Availability

The data supporting the findings of this study are sensitive data, therefore there are access restrictions that prevent its public availability. However, these data can be provided by the corresponding author upon reasonable request, with prior permission from a research ethics committee.

## Abbreviations

aOR: Adjusted Odds Ratio
ARD: Acure Respiratory Distress
CI: Confidence Intervals
HDI: Human Development Index
ICU: Intensive Care Unit
RAS: Renin-Angiotensin System
SIM: Mortality Information System / *Sistema de Informação sobre Mortalidade*
SINASC: Live Births Information System / *Sistema de Informação sobre Nascidos Vivos*
SIVEP: Acute Respiratory Syndrome Surveillance System / *Sistema de Vigilância Epidemiológica da Síndrome Respiratória Aguda Grave*

## References

[1] United Nations Development Programme | UNDP. Coronavirus, COVID-19 pandemic. An integrated global response is an investment in our future. Available at: https://www.undp.org/coronavirus. Accessed 17 Apr 2022.

[2] Mas-Coma S, Jones MK, Marty AM. COVID-19 and globalization. One Health. 2020;9:100132. 10.1016/j.onehlt.2020.100132. 10.1016/j.onehlt.2020.100132 PMC7184197 32368611

[3] Worldmeters. Coronavirus Update (Live) COVID-19 Coronavirus Pandemic. 2022. Available at: https://www.worldometers.info/coronavirus/. Accessed 30 Dec 2022.

[4] World Health Organization | WHO. Impact of COVID-19 on people’s livelihoods, their health and our food systems - Joint statement by ILO, FAO, IFAD and WHO. 2020. Available at: https://www.who.int/news/item/13-10-2020-impact-of-covid-19-on-people’s-livelihoods-their-health-and-our-food-systems. Accessed 17 Apr 2022.

[5] World Health Organization | WHO. The impact of COVID-19 on global health goals. 2021. Available at: https://www.who.int/news-room/spotlight/the-impact-of-covid-19-on-global-health-goals. Accessed 17 Apr 2022.

[6] Silasi M, Cardenas I, Kwon JY, Racicot K, Aldo P, Mor G. Viral Infections During Pregnancy. Am J Reprod Immunol. 2015;73. 10.1111/aji.12355 PMC4610031 25582523

[7] World Health Organization | WHO. Conflict, climate crisis and COVID-19 pose great threats to the health of women and children. 2020. Available at: https://www.who.int/news/item/25-09-2020-conflict-climate-crisis-and-covid-19-pose-great-threats-to-the-health-of-women-and-children. Accessed 18 Apr 2022.

[8] Organización Panamericana de la Salud | PAHO. Alerta Epidemiológica: COVID-19 durante el embarazo- 13 de agosto de 2020 - OPS/OMS. 2020. Available at: https://www.paho.org/es/documentos/alerta-epidemiologica-covid-19-durante-embarazo-13-agosto-2020. Accessed 12 Oct 2020.

[9] Brasil. Ministério da Saúde. Secretaria de Vigilância em Saúde. Painel de casos de doença pelo coronavírus 2019 (COVID-19). 2021. Available at: https://covid.saude.gov.br/. Accessed 24 Apr 2021.

[10] Brasil. Ministério da Saúde. openDATASUS. 2022. Available at: https://opendatasus.saude.gov.br/organization/ministerio-da-saude. Accessed 31 Dec 2022.

[11] Souza ASR, Amorim MMR. Maternal mortality by COVID-19 in Brazil. Revista Brasileira de Saúde Materno Infantil. 2021;21 suppl 1:253–6.

[12] Robinson GE. Pregnancy loss. Best Pract Res Clin Obstet Gynaecol. 2014;28:169–78. 10.1016/j.bpobgyn.2013.08.012 24047642

[13] Instituto Brasileiro de Geografia e Estatística IBGE. IBGE Cidades. 2019. Available at: https://cidades.ibge.gov.br/brasil/ba/panorama. Accessed 12 Dec 2019.

[14] Brasil. Ministério da Saúde. Secretaria de Vigilância em Saúde. Secretaria de Atenção à Saúde. Manual de vigilância do óbito infantil e fetal e do Comitê de Prevenção do Óbito Infantil e Fetal. 2nd edition. Brasília, DF; 2009. Available at: https://www.gov.br/saude/pt-br/assuntos/saude-de-a-a-z/s/saude-da-crianca/publicacoes/manual-de-vigilancia-do-obito-infantil-e-fetal-e-do-comite-de-prevencao-do-obito-infantil-e-fetal/@@download/file. Accessed 27 Apr 2023.

[15] Orellana J, Jacques N, Leventhal DGP, Marrero L, Morón-Duarte LS (2022). Excess maternal mortality in Brazil: Regional inequalities and trajectories during the COVID-19 epidemic. PLoS ONE.

[16] Gonçalves BMM, Franco RPV, Rodrigues AS (2021). Maternal mortality associated with COVID-19 in Brazil in 2020 and 2021: comparison with non-pregnant women and men. PLoS ONE.

[17] Brasil. Ministério da Saúde. Secretaria de Vigilância em Saúde. Departamento de Vigilância das Doenças Transmissíveis. Protocolo de tratamento de Influenza: 2017 [recurso eletrônico]. 2018;1:1–49. Available at: https://bvsms.saude.gov.br/bvs/publicacoes/protocolo_tratamento_influenza_2017.pdf. Accessed 27 Apr 2023.

[18] Brasil. Ministério da Saúde. Secretaria de Vigilância em Saúde e Ambiente. Sistemas de Informação em Saúde. 2021. Available at: https://www.gov.br/saude/pt-br/composicao/svs/vigilancia-de-doencas-cronicas-nao-transmissiveis/sistemas-de-informacao-em-saude. Accessed 30 Dec 2022.

[19] Brasil. Ministério da Saúde. Secretaria de Vigilância em Saúde. Sistema de Informações sobre Mortalidade-SIM Consolidação da base de dados de 2011. Brasília, DF. 2011. Available at: http://tabnet.datasus.gov.br/cgi/sim/Consolida_Sim_2011.pdf. Accessed 27 Apr 2023.

[20] de Morais RM, Costa AL (2017). Uma avaliação do Sistema de Informações sobre Mortalidade. Saúde em Debate.

[21] Szwarcwald  CL,  do Carmo Leal  M, Esteves-Pereira  AP, da Silva de Almeida  W, de Frias  PG, Damacena  GN (2019). Evaluation of data from the brazilian information system on live births (SINASC). Cad Saude Publica.

[22] Mathers CD, Fat DM, Inoue M, Rao C, Lopez AD. Counting the dead and what they died from: an assessment of the global status of cause of death data. Bull World Health Organ. 2005;83(3):171–7. PMC2624200 15798840

[23] Brasil. Ministério da Saúde. Secretaria de Vigilância em Saúde. Guia de Vigilância Epidemiológica: Emergência de Saúde Pública de Importância Nacional pela COVID-19. 3rd edition. Brasília; 2022. Available at: https://www.gov.br/saude/pt-br/coronavirus/publicacoes-tecnicas/guias-e-planos/guia-de-vigilancia-epidemiologica-covid-19/view. Accessed 27 Apr 2023.

[24] Enamorado, Ted, Benjamin Fifield, and Kosuke Imai. 2017. fastLink: Fast Probabilistic Record Linkage with Missing Data. Version 0.6. Available at: https://github.com/kosukeimai/fastLink. Accessed 27 Apr 2023.

[25] R Foundation for Statistical Computing. R: A language and environment for statistical computing. 2021;3. Available at: https://www.r-project.org/. Accessed 27 Apr 2023.

[26] von Elm E, Altman DG, Egger M, Pocock SJ, Gøtzsche PC, Vandenbroucke JP (2014). The Strengthening the Reporting of Observational Studies in Epidemiology (STROBE) Statement: guidelines for reporting observational studies. Int J Surg.

[27] Sharma A. Respiratory distress. In: Kliegman RM, Lye PS, Bordini BJ, Toth H, Basel D, editors. Nelson Pediatric Symptom-Based Diagnosis. Elsevier; 2018. p. 39–60.e1. 10.1016/B978-0-323-39956-2.00003-0.

[28] Cole DE, Taylor TL, McCullough DM, Shoff CT, Derdak S (2005). Acute respiratory distress syndrome in pregnancy. Crit Care Med.

[29] Phoswa WN, Khaliq OP (2020). Is pregnancy a risk factor of COVID-19?. Eur J Obstet Gynecol Reprod Biol.

[30] Hegewald MJ, Crapo RO (2011). Respiratory physiology in pregnancy. Clin Chest Med.

[31] Wong SF, Chow KM, de Swiet M (2003). Severe acute respiratory syndrome and pregnancy. BJOG.

[32] Abbas AM, Ahmed OA, Shaltout AS (2020). COVID-19 and maternal pre-eclampsia; a synopsis. Scand J Immunol.

[33] Zheng YY, Ma YT, Zhang JY, Xie X (2020). COVID-19 and the cardiovascular system. Nat Rev Cardiol.

[34] Papageorghiou AT, Deruelle P, Gunier RB, Rauch S, García-May PK, Mhatre M (2021). Preeclampsia and COVID-19: results from the INTERCOVID prospective longitudinal study. Am J Obstet Gynecol.

[35] Harmon QE, Huang L, Umbach DM, KlungsØyr K, Engel SM, Magnus P (2015). Risk of fetal death with preeclampsia. Obstet Gynecol.

[36] Thornton P, Douglas J (2010). Coagulation in pregnancy. Best Pract Res Clin Obstet Gynaecol.

[37] Hanff TC, Mohareb AM, Giri J, Cohen JB, Chirinos JA (2020). Thrombosis in COVID-19. Am J Hematol.

[38] Lazzaroni MG, Piantoni S, Masneri S, Garrafa E, Martini G, Tincani A (2021). Coagulation dysfunction in COVID-19: the interplay between inflammation, viral infection and the coagulation system. Blood Rev.

[39] Mongula JE, Frenken MWE, van Lijnschoten G, Arents NLA, de Wit-Zuurendonk LD, Schimmel-de Kok APA (2020). COVID-19 during pregnancy: non-reassuring fetal heart rate, placental pathology and coagulopathy. Ultrasound Obstet Gynecol.

[40] Seymen CM (2021). Being pregnant in the COVID-19 pandemic: effects on the placenta in all aspects. J Med Virol.

[41] Konstantinidou AE, Angelidou S, Havaki S, Paparizou K, Spanakis N, Chatzakis C (2022). Stillbirth due to SARS-CoV-2 placentitis without evidence of intrauterine transmission to fetus: association with maternal risk factors. Ultrasound Obstet Gynecol.

[42] Stenton S, McPartland J, Shukla R, Turner K, Marton T, Hargitai B (2022). SARS-COV2 placentitis and pregnancy outcome: a multicentre experience during the Alpha and early Delta waves of coronavirus pandemic in England. EClinicalMedicine.

[43] Schwartz DA, Mulkey SB, Roberts DJ (2023). SARS-CoV-2 placentitis, stillbirth, and maternal COVID-19 vaccination: clinical-pathologic correlations. Am J Obstet Gynecol.

[44] Smith ER, Oakley E, Grandner GW Perinatal COVID PMA Study Collaborators, et al. Adverse maternal, fetal, and newborn outcomes among pregnant women with SARS-CoV-2 infection: an individual participant data meta-analysis. BMJ Global Health. 2023;8:e009495. 10.1136/bmjgh-2022-009495 PMC9895919 36646475

[45] Figueiro-Filho EA, Yudin M, Farine D. COVID-19 during pregnancy: an overview of maternal characteristics, clinical symptoms, maternal and neonatal outcomes of 10,996 cases described in 15 countries. J Perinat Med. 2020;48(9):900–11. 10.1515/jpm-2020-0364. 10.1515/jpm-2020-0364 33001856

[46] Mirbeyk M, Saghazadeh A, Rezaei N. A systematic review of pregnant women with COVID-19 and their neonates. Arch Gynecol Obstet. 2021;304:5–38. 10.1007/s00404-021-06049-z. 10.1007/s00404-021-06049-z PMC8017514 33797605

[47] Örtqvist AK, Magnus MC, Aabakke AJM, Urhoj SK, Vinkel Hansen A, Nybo Andersen AM, Krebs L, Pettersson K, Håberg SE, Stephansson O. Severe COVID-19 during pregnancy in Sweden, Norway, and Denmark. Acta Obstet Gynecol Scand. 2023. 10.1111/aogs.14552. 10.1111/aogs.14552 PMC10201957 36928990

[48] Vousden N, Ramakrishnan R, Bunch K, Morris E, Simpson N, Gale C (2022). Management and implications of severe COVID-19 in pregnancy in the UK: data from the UK obstetric surveillance system national cohort. Acta Obstet Gynecol Scand.

[49] Pinheiro RL, Areia AL, Pinto AM, Donato H (2019). Advanced maternal age: adverse outcomes of pregnancy a meta-analysis. Acta Med Port.

[50] Kayem G, Lecarpentier E, Deruelle P, Bretelle F, Azria E, Blanc J (2020). A snapshot of the Covid-19 pandemic among pregnant women in France. J Gynecol Obstet Hum Reprod.

[51] Adeyinka DA, Olakunde BO, Muhajarine N (2019). Evidence of health inequity in child survival: spatial and Bayesian network analyses of stillbirth rates in 194 countries. Scientific Reports.

[52] Cheong-See F, Schuit E, Arroyo-Manzano D, Khalil A, Barrett J, Joseph KS (2016). Prospective risk of stillbirth and neonatal complications in twin pregnancies: systematic review and meta-analysis. BMJ.

[53] Mizuno R (2000). The male/female ratio of fetal deaths and births in Japan. Lancet.

[54] Aghai ZH, Goudar SS, Patel A, Saleem S, Dhaded SM, Kavi A (2020). Gender variations in neonatal and early infant mortality in India and Pakistan: a secondary analysis from the global network maternal newborn health registry. Reprod Health.

[55] Mcmillen MM (1979). Differential mortality by sex in fetal and neonatal deaths. Science.

[56] Bahia. Comissão Intergestores Bipartite. Resolução CIB no 077/2021. Aprova as propostas da 15a Reunião Extraordinária da CIB, referentes à atualização da vacinação dos grupos prioritários. Salvador; 2021. Available at: http://www5.saude.ba.gov.br/portalcib/index.php?option=com_content&view=article&id=660&Itemid=183. Accessed 27 Apr 2023.

[57] Brasil. Governo Federal. Governo recomenda vacinação contra Covid-19 em gestantes e puérperas sem comorbidades. 2021. https://www.gov.br/pt-br/noticias/saude-e-vigilancia-sanitaria/2021/07/governo-recomenda-vacinacao-contra-covid-19-em-gestantes-e-puerperas-sem-comorbidades. Accessed 18 Mar 2023.

