# Supplementary material for: Fetal death as an outcome of acute respiratory distress in pregnancy, during the COVID-19 pandemic: a population-based cohort study in Bahia, Brazil
Source: BMC Pregnancy Childbirth. 2023 May 5;23:320. doi: 10.1186/s12884-023-05601-w (PMC10161155; doi:10.1186/s12884-023-05601-w)
Supplement: Supplementary file 1 — Additional file 1. [file 12884_2023_5601_MOESM1_ESM.docx]

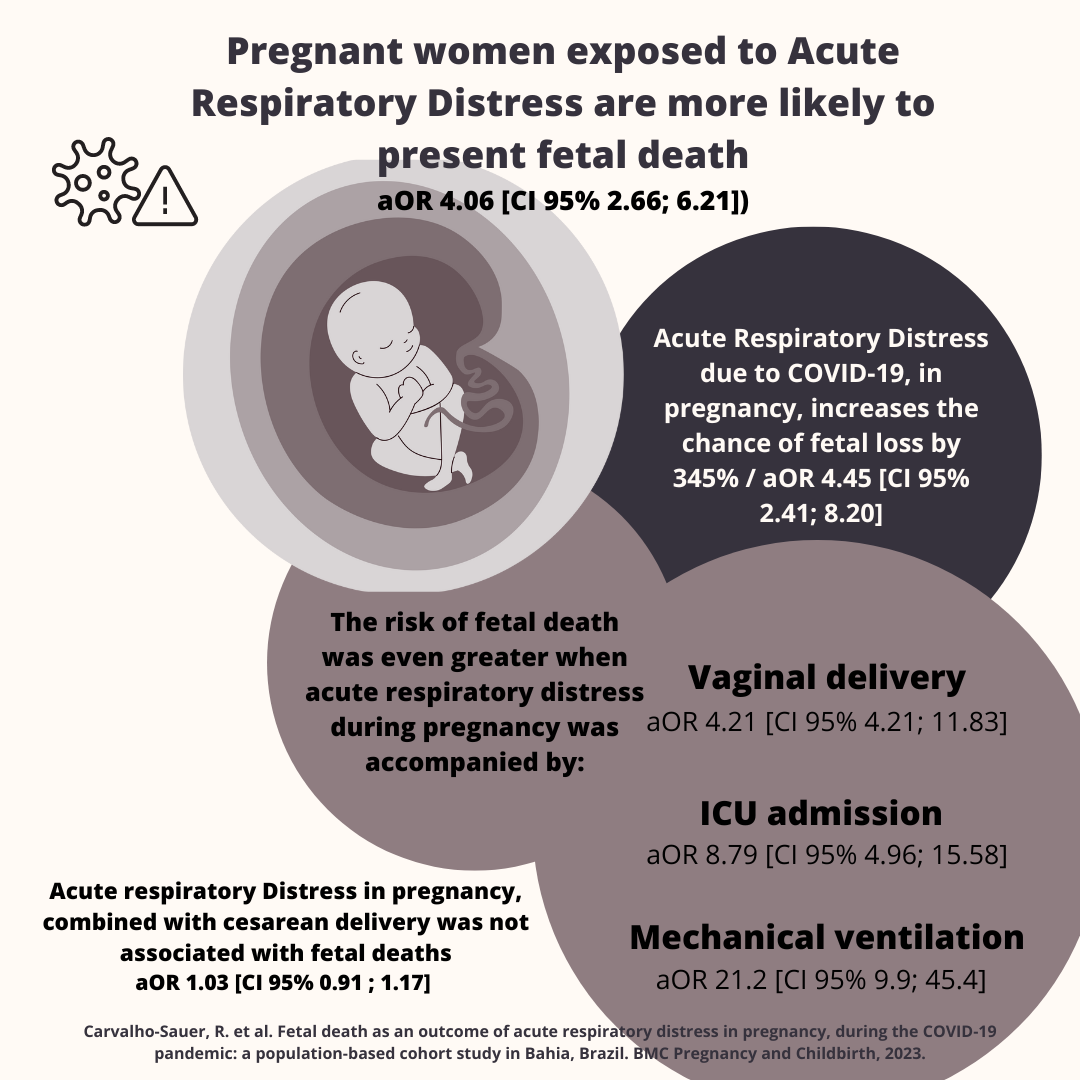


**Supplementary Infographic.** Main results of the study 'Fetal death as an outcome of acute respiratory distress in pregnancy, during the COVID-19 pandemic: a population-based cohort study in Bahia, Brazil'**.**
