# Supplementary material for: Fetal death as an outcome of acute respiratory distress in pregnancy, during the COVID-19 pandemic: a population-based cohort study in Bahia, Brazil
Source: BMC Pregnancy Childbirth. 2023 May 5;23:320. doi: 10.1186/s12884-023-05601-w (PMC10161155; doi:10.1186/s12884-023-05601-w)
Supplement: Supplementary file 2 — Additional file 2. [file 12884_2023_5601_MOESM2_ESM.docx]

**STROBE—checklist of “Fetal death as an outcome of acute respiratory distress in pregnancy, during the COVID-19 pandemic: a population-based cohort study in Bahia, Brazil”.**

|  | Item No. | Recommendation | Page  No. | Relevant text from manuscript |
| --- | --- | --- | --- | --- |
| **Title and abstract** | 1 | (*a*) Indicate the study’s design with a commonly used term in the title or the abstract | 1 | Title: Fetal death as an outcome of acute respiratory distress in pregnancy, during the COVID-19 pandemic: a population-based cohort study in Bahia, Brazil. |
|  |  | (*b*) Provide in the abstract an informative and balanced summary of what was done and what was found | 3 | Methods: (…) We linked administrative data (under mandatory registration) on live births, fetal deaths, and acute respiratory syndrome, using a probabilistic linkage method, and analyzed them with multivariable logistic regression models.  Results: (…)We found four times higher chance of fetal death in women with ARD during pregnancy, of all etiologies (adjusted odds ratio [aOR] 4.06 confidence interval [CI] 95% 2.66; 6.21), and due to SARS-CoV-2 (aOR 4.45 CI 95% 2.41; 8.20). The risk of fetal death increased more when ARD in pregnancy was accompanied by vaginal delivery (aOR 7.06 CI 95% 4.21; 11.83), or admission to Intensive Care Unit (aOR 8.79 CI 95% 4.96; 15.58), or use of invasive mechanical ventilation (aOR 21.22 CI 95% 9.93; 45.36). |
| Introduction | | | |  |
| Background/rationale | 2 | Explain the scientific background and rationale for the investigation being reported | 5 - 6 | (…) Far beyond a health tragedy, the disruptive effect of the COVID-19 pandemic on the social and economic structures worldwide has disproportionately affected the most vulnerable populations.  (…) Furthermore, since the onset of the COVID-19 pandemic, Brazil has recorded an unprecedented number of hospitalizations of pregnant women due to acute respiratory distress (ARD). Epidemiological surveillance data showed that in 2020 and 2021, there were 28,238 reports of ARD in pregnant women in Brazil, compared to 2,252 reported in 2018-2019. |
| Objectives | 3 | State specific objectives, including any prespecified hypotheses | 6 | Fetal loss is one of the most serious adverse outcomes of pregnancy [12]. So, we aimed to assess the chance of fetal death associated with acute respiratory distress during pregnancy, in the context of the COVID-19 pandemic in Bahia, Brazil. |
| Methods | | | |  |
| Study design | 4 | Present key elements of study design early in the paper | 6 | We performed a retrospective cohort study (…) |
| Setting | 5 | Describe the setting, locations, and relevant dates, including periods of recruitment, exposure, follow-up, and data collection | 6 - 8 | We performed a retrospective cohort study, using administrative data on live births, fetal deaths, and ARD in pregnant women, from January 2019 to June 2021, in Bahia, a state with 14,985,284 inhabitants located in the Northeast region of Brazil [13].  The exposed group consisted of women who had a pregnancy ending in 2020 or 2021, and who had a history of hospitalization due to acute respiratory distress during this pregnancy, consistent with respiratory viruses, that is, a combination of flu-like symptoms such as fever and cough with acute respiratory distress syndrome, and/or dyspnea, and/or O2 saturation <95% in ambient air, and/or cyanosis, and/or hypotension and/or acute breathing insufficiency, regardless of its etiology [15].  The unexposed group was women who had a pregnancy ending in 2019 (pre-pandemic period) and did not undergo a hospital stay due to respiratory distress, because they were pregnancies free of the possible effects of SARS-CoV-2 infection and the environmental peculiarities of the COVID-19 pandemic period.  We used data from robust health information systems [16], such as the Live Births Information System / Sistema de Informação sobre Nascidos Vivos (SINASC); the Mortality Information System / Sistema de Informação sobre Mortalidade (SIM); and the Acute Respiratory Syndrome Surveillance System / Sistema de Vigilância Epidemiológica da Síndrome Respiratória Aguda Grave (SIVEP). |
| Participants | 6 | (*a*) *Cohort study*—Give the eligibility criteria, and the sources and methods of selection of participants. Describe methods of follow-up  *Case-control study*—Give the eligibility criteria, and the sources and methods of case ascertainment and control selection. Give the rationale for the choice of cases and controls  *Cross-sectional study*—Give the eligibility criteria, and the sources and methods of selection of participants | 6 | The population comprised women with ≥ 20 weeks of pregnancy, residents in Bahia state. Therefore, the outcome "fetal deaths" in this study included spontaneous intrauterine death of fetuses at or after 20 gestational weeks (or weighing ≥ 500g, or length ≥ 25cm, if unknown gestational age [14]).  As this study focuses on the association between ARD during pregnancy and fetal death, we chose not to include fetal deaths that occurred concurrently with maternal deaths, as it would be more difficult to determine clearly whether fetal death occurred before or after maternal death, in these cases. |
|  |  | (*b*) *Cohort study*—For matched studies, give matching criteria and number of exposed and unexposed  *Case-control study*—For matched studies, give matching criteria and the number of controls per case |  | Not applicable |
| Variables | 7 | Clearly define all outcomes, exposures, predictors, potential confounders, and effect modifiers. Give diagnostic criteria, if applicable | 6 - 9 | (…) the outcome "fetal deaths" in this study included spontaneous intrauterine death of fetuses at or after 20 gestational weeks (or weighing ≥ 500g, or length ≥ 25cm, if unknown gestational age [14]).  (…)The exposed group consisted of women who had a pregnancy ending in 2020 or 2021, and who had a history of hospitalization due to acute respiratory distress during this pregnancy, consistent with respiratory viruses, that is, a combination of flu-like symptoms such as fever and cough with acute respiratory distress syndrome, and/or dyspnea, and/or O2 saturation <95% in ambient air, and/or cyanosis, and/or hypotension and/or acute breathing insufficiency, regardless of its etiology. (…)  To verify the risk of fetal death after ARD during pregnancy, as well as to identify potential confounders, we applied a bivariate logistic regression model. Those variables that showed association with statistical significance in bivariate analysis were included in a multiple model, to estimate the adjusted Odds Ratio (OR) with 95% confidence intervals (CI).  (…) acute respiratory distress during this pregnancy, consistent with respiratory viruses, that is, a combination of flu-like symptoms such as fever and cough with acute respiratory distress syndrome, and/or dyspnea, and/or O2 saturation <95% in ambient air, and/or cyanosis, and/or hypotension and/or acute breathing insufficiency, regardless of its etiology [15]. (…)  (…) And to quantify the risk of fetal death after ARD in pregnancy, specifically caused by laboratory-confirmed COVID-19, this same analysis was repeated, keeping in the exposed group only participants with reverse-transcriptase polymerase chain reaction (RT-PCR), antigen detection, or IgM serology tests positive for SARS-CoV-2 infection. (…)  The study variables, related to the sociodemographic characteristics of the participants, were: Age group, in years (19 or less , 20 - 35 , 36 or more); Education, in years (0 - 3 , 4 - 7 , 8 - 11 , 12 or more); Race Color (White , Black , Mixed , Oriental , Indigenous); Marital Status (Single or Divorced or Widow , Married or Stable Union) and Human Development Index of municipality of residence (High or Very High , Medium , Low or Very Low).  The variables related to the characteristics of the pregnancy were: Fetal death (Yes , No); Acute respiratory Distress during pregnancy (Yes , No); Type of pregnancy (Single fetus , Twin fetus or more); Fetus sex (Male , Female); Parity (1st pregnancy , 2nd or more); Type of delivery (Vaginal , Cesarean); Congenital anomaly (Yes , No); and Gestational age, in weeks (1st quartile , median , 3rd quartile). |
| Data sources/ measurement | 8* | For each variable of interest, give sources of data and details of methods of assessment (measurement). Describe comparability of assessment methods if there is more than one group | 7 - 8 | (…) SINASC and SIM contain all national records of births and deaths, respectively, and their coverages are approximately 100% [17–19] (…)  (…) SIVEP contains all national records of people who present ARD consistent with respiratory viruses, reported by health services. But, if it occurs outside a health service, such as death from ARD at home, epidemiological surveillance teams carry out an epidemiological investigation and reporting at SIVEP [15, 21]. (…) |
| Bias | 9 | Describe any efforts to address potential sources of bias |  | To minimize the possibility of failures in the linkage procedure, all pairs were manually reviewed by the authors, with verification of the residential address, thus avoiding the linking of records of homonyms. Regarding the diagnostic criterion for ARD, sensitivity tests were applied, including only cases with laboratory diagnosis, as described below:  (…) Records with at least 85% of similarity were filtered. So, we performed a manual review, with a comparison of home addresses, to confirm only valid pairs. (…)  And to quantify the risk of fetal death after ARD in pregnancy, specifically caused by laboratory-confirmed COVID-19, this same analysis was repeated, keeping in the exposed group only participants with reverse-transcriptase polymerase chain reaction (RT-PCR), antigen detection, or IgM serology tests positive for SARS-CoV-2 infection. |
| Study size | 10 | Explain how the study size was arrived at | 6 | In this study, we did not work with sampling, as this was a population-based study.  (…) We performed a population-based retrospective cohort study (…) |

| Quantitative variables | 11 | Explain how quantitative variables were handled in the analyses. If applicable, describe which groupings were chosen and why | 9 - 10 | (…) The sociodemographic and gestational characteristics of the participants (age group, education, race/color, marital status, Human Development Index (HDI) of the municipality of residence, fetal death as outcome of pregnancy, type of pregnancy, fetus sex, parity, type of delivery and congenital anomaly of fetus) were described by the absolute and relative frequencies of each variable. Two-tailed Z-test, for comparing two proportions, was performed to identify divergences in gestational characteristics between women with and without a history of ARD during pregnancy. We considered a significance level of 5%.  To verify the risk of fetal death after ARD during pregnancy, as well as to identify potential confounders, we applied a bivariate logistic regression model. (…) | |
| --- | --- | --- | --- | --- | --- |
| Statistical methods | 12 | (*a*) Describe all statistical methods, including those used to control for confounding | 9 - 10 | Those variables that showed association with statistical significance in bivariate analysis were included in a multiple model, to estimate the adjusted Odds Ratio (OR) with 95% confidence intervals (CI). (…) | |
|  |  | (*b*) Describe any methods used to examine subgroups and interactions | 9 - 10 | And to quantify the risk of fetal death after ARD in pregnancy, specifically caused by laboratory-confirmed COVID-19, this same analysis was repeated, keeping in the exposed group only participants with reverse-transcriptase polymerase chain reaction (RT-PCR), antigen detection, or IgM serology tests positive for SARS-CoV-2 infection. We also assessed the risk of fetal death after ARD in pregnancy with Intensive Care Unit (ICU) admission, with invasive mechanical ventilation, and with vaginal or cesarean delivery. | |
|  |  | (*c*) Explain how missing data were addressed |  |  | |
|  |  | (*d*) *Cohort study*—If applicable, explain how loss to follow-up was addressed  *Case-control study*—If applicable, explain how matching of cases and controls was addressed  *Cross-sectional study*—If applicable, describe analytical methods taking account of sampling strategy | 9 – 10 | The exposure and outcome variables had 100% completeness. The percentage of missing data in covariates (sociodemographic and gestational characteristics) was less than 10% for most of them. We did not consider that missing data interfered with the results of this study. We presented in Tables 1 and 2 the values of existing data in each category of the variables. | |
|  |  | (*e*) Describe any sensitivity analyses | 9 - 10 | (…) to quantify the risk of fetal death after ARD in pregnancy, specifically caused by laboratory-confirmed COVID-19, this same analysis was repeated, keeping in the exposed group only participants with reverse-transcriptase polymerase chain reaction (RT-PCR), antigen detection, or IgM serology tests positive for SARS-CoV-2 infection. | |
| Results | | | | |  |
| Participants | 13* | (a) Report numbers of individuals at each stage of study—eg numbers potentially eligible, examined for eligibility, confirmed eligible, included in the study, completing follow-up, and analysed | 10 | After probabilistic linkage of data from SINASC, SIM and SIVEP, 765 participants were identified as pregnant women who had ARD during pregnancy (throughout the COVID-19 pandemic, Jan 2020 – Jun 2021), and another 200,214 who did not (Jan to Dec 2019). The 32 participants who had ARD during pregnancy in the pre-pandemic period (2019),; the 6 (six) fetal deaths that occurred concurrently with maternal deaths, and the 284,862 women who did not have ARD during pregnancy in the COVID-19 pandemic period (until Jun, 2021) were excluded (Figure 1). | |
|  |  | (b) Give reasons for non-participation at each stage | 7 | During the COVID-19 pandemic, there was a significant increase in maternal mortality from several causes in Brazil [15, 16]. As this study focuses on the association between ARD during pregnancy and fetal death, we chose not to include fetal deaths that occurred concurrently with maternal deaths, as it would be more difficult to determine clearly whether fetal death occurred before or after maternal death, in these cases. | |
|  |  | (c) Consider use of a flow diagram | 12 | See Figure 1 | |
| Descriptive data | 14* | (a) Give characteristics of study participants (eg demographic, clinical, social) and information on exposures and potential confounders | 13; 15 | See Table 1 and Table 2 | |
|  |  | (b) Indicate number of participants with missing data for each variable of interest | 13; 15 | See Table 1 and Table 2 | |
|  |  | © *Cohort study*—Summarise follow-up time (eg, average and total amount) | 13; 15 | See Table 1 and Table 2 | |
| Outcome data | 15* | *Cohort study*—Report numbers of outcome events or summary measures over time | 15 | See Table 2 | |
|  |  | *Case-control study—*Report numbers in each exposure category, or summary measures of exposure | - | Not applicable | |
|  |  | *Cross-sectional study—*Report numbers of outcome events or summary measures | - | Not applicable | |
| Main results | 16 | (*a*) Give unadjusted estimates and, if applicable, confounder-adjusted estimates and their precision (eg, 95% confidence interval). Make clear which confounders were adjusted for and why they were included | 16 | See Table 3 | |
|  |  | (*b*) Report category boundaries when continuous variables were categorized | - | Not applicable | |
|  |  | (*c*) If relevant, consider translating estimates of relative risk into absolute risk for a meaningful time period | - | Not applicable | |

| Other analyses | 17 | Report other analyses done—eg analyses of subgroups and interactions, and sensitivity analyses | 18 | See Table 4 |
| --- | --- | --- | --- | --- |
| Discussion | | | | |
| Key results | 18 | Summarise key results with reference to study objectives | 18 | (…) This study showed that during the first two years of the COVID-19 pandemic (2020-2021) SARS-CoV-2 virus was the main cause of ARD in pregnant women from Bahia, Brazil. (…)  (…) The chance of fetal death was more than four times higher among the exposed when compared to the unexposed group, either for all etiologies or specifically when the causative agent of ARD was the SARS-CoV-2 virus. And critical forms of ARD, requiring artificial respiration support, increased the chance of this outcome by up to 21 times. (…) |
| Limitations | 19 | Discuss limitations of the study, taking into account sources of potential bias or imprecision. Discuss both direction and magnitude of any potential bias | 22 | Limitations of this study include the use of administrative data, the use of preliminary data for 2021, the possibility of errors in the linkage of the databases, lack of knowledge regarding vaccine status, virus variants, maternal comorbidities and potential underreporting. |
| Interpretation | 20 | Give a cautious overall interpretation of results considering objectives, limitations, multiplicity of analyses, results from similar studies, and other relevant evidence | 22 | Our results must be interpreted considering their strengths and limitations. (…) |
| Generalisability | 21 | Discuss the generalisability (external validity) of the study results | 23 | (…) study to estimate the risk of fetal deaths associated to ARD in pregnancy, in the context of COVID-19 pandemic, in a middle-income country, and in a statewide population-based cohort |
| Other information | |  | | |
| Funding | 22 | Give the source of funding and the role of the funders for the present study and, if applicable, for the original study on which the present article is based | 26 | ESP is funded by the Wellcome Trust. The funder had no role in the study design or conduct, data collection, management, analysis, interpretation, or writing of the report. |

*Give information separately for cases and controls in case-control studies and, if applicable, for exposed and unexposed groups in cohort and cross-sectional studies.
